# Humor in Dark Personalities: An Empirical Study on the Link Between Four Humor Styles and the Distinct Subfactors of Psychopathy and Narcissism

**DOI:** 10.3389/fpsyg.2021.548450

**Published:** 2021-04-12

**Authors:** Jill Lobbestael, Vanessa Lea Freund

**Affiliations:** Department of Clinical Psychological Science, Faculty of Psychology and Neuroscience, Maastricht University, Maastricht, Netherlands

**Keywords:** humor, narcissism, psychopathy, Triarchic model psychopathy, subfactors

## Abstract

**Background:**

Humor is a main ingredient of interpersonal relationships. Two sets of psychopathological traits known for their devastating impact on interpersonal relationships are psychopathy and narcissism. The current study was developed to provide a fine-grained analysis of the relationship between four humor styles (affiliative, self-enhancing, self-defeating, and aggressive) and both psychopathic and narcissistic traits. Specifically, it addresses how humor styles relate to the three psychopathy subfactors (following the triarchic model) and four subfactors of grandiose narcissism.

**Method:**

Self-report measures in a non-clinical male sample *N* = 177.

**Results:**

Multiple regression analyses revealed psychopathic and narcissistic traits’ relation to using both benign and injurious humor. Subfactor analyses showed that aggressive and self-defeating humor were mostly associated with impulsivity and entitlement, while dominance levels actuated the use of humor to cope with stress. The cold-heartedness component of psychopathy proved to be particularly humorless, setting it aside as a distinctively disturbing psychopathic subfactor.

**Conclusions and implications:**

Humor strongly colors the interpersonal style of both psychopathic and narcissistic personalities. Differential components of both personality types inform on the possible underlying motivations that drive the use of distinct styles of humor. This implies that psychopathic and narcissistic traits could potentially be lowered through the alternation of humor styles.

## Introduction

Humor is an essential component of interpersonal communication. Earlier research framed humor as an adaptive trait enhancing one’s physical and psychological health (Lefcourt, 2001). Later, a more nuanced model outlined two central humor dimensions. The first dimension determines that humor can be either benign or injurious; while the second dimension differentiates the potential of humor to enhance the relationship with either others or the self. The interaction of these two dimensions leads to four distinct humor styles: affiliative humor (friendly humor to enhance interpersonal bounds), self-enhancing humor (benignly enhancing the self), self-defeating humor (amusing others through self-disparaging), and aggressive humor (putting others down to enhance oneself, e.g., sarcastically) (Martin et al., 2003). Studies empirically linked these humor styles to differential constructs, evidencing their distinctiveness. Affiliative and self-enhancing humor styles, for example, have been shown to relate to positive outcomes like self-esteem (Galloway, 2010), mental toughness (Veselka et al., 2010), and social support (DeLongis and Holtzman, 2005). In contrast, self-defeating humor has been linked to social isolation, emotional distress, and hostility, while aggressive humor is related to externalizing problems (Dozois et al., 2013).

The current study focusses on humor at its relationship with two personality constellations that strongly impact interpersonal relationships: psychopathy and narcissism. Psychopathy is characterized by deficits in emotional function and antisocial behavior. Narcissism primarily reflects a cognitive-affective preoccupation with the self. Communalities in both of these sets of traits are a self-centered focus, and the tendency to place one’s needs above those of others. Consequently, familial, parenting, romantic, and professional relationships of people with increased levels of psychopathy or narcissism are often characterized by high stress levels, low commitment, conflicts, and counter productivity (e.g., Campbell and Foster, 2002; Penney and Spector, 2002; Fite et al., 2008; Campbell et al., 2011). Excessive levels of both psychopathic and narcissistic traits are often found in forensic and criminal settings (Spaans et al., 2017; Lambe et al., 2018), and are related to therapy-resistance and increased levels of re-offending (e.g., Bennett, 2015; Shepherd and Strand, 2016). However, research has also shown that psychopathic and narcissistic traits do not uniquely express in clinical or forensic subgroups. Rather, they reflect dimensional constructs with varying levels in the general population (Guay et al., 2007), which is also the target group of the current study. Importantly, psychopathy and narcissism should not be seen as unitary but rather as multi-faceted concepts. For psychopathy, there is good support for a triarchic model (Patrick et al., 2009; Drislane et al., 2014), which identifies boldness (dominance, emotional stability, and venturesomeness), disinhibition (deficient inhibitory control), and meanness (callousness and aggressive resource seeking) as core distinct psychopathic features. Within narcissism, mostly four subfactors are differentiated: superiority/arrogance, self-absorption/self-administration, exploitativeness/entitlement, and leadership/authority. While the latter subfactor can be more adaptive in nature, the other narcissistic traits largely impact social relationships maladaptively (Miller and Campbell, 2008; Pincus and Lukowitsky, 2010).

Previous empirical studies on humor styles and psychopathic and narcissistic traits mostly supported the link between these personality constellations and the negative humor styles (i.e., aggressive and self-defeating). Additionally, narcissistic traits have been linked to both positive humor styles (Veselka et al., 2010; Besser and Zeigler-Hill, 2011; Zeigler-Hill and Besser, 2011; Martin et al., 2012; Masui et al., 2013). Aside from this, hypotheses on which humor styles would correlate to psychopathic and narcissistic traits can be based on the socioanalytical theory (Hogan and Foster, 2016), which describes personality traits primarily in terms of individual differences in expressing motives to either *getting ahead* or *getting along*. A similar distinction is between an *agentic* versus a *communal* focus, with the first referring to power and achievement, and the latter to one’s sense of belonging and relationships with others (Woike, 1994). Both psychopathy and narcissism are primarily related to aiming to get ahead and to an agentic focus (Campbell et al., 2007; Jones and Paulhus, 2011; Rauthmann and Kolar, 2013; Grijalva and Zhang, 2016; Jonason and Fletcher, 2018). Humor can be considered as a form of an assertive self-presentation technique that people with narcissistic and psychopathic traits use to maintain and enhance their reputation when interacting with others. Such reputation improvement can be established by enhancing one’s own status, that is, by getting ahead or by lowering the others’ statuses. Therefore, both psychopathic and narcissistic traits can be assumed to predispose to augmenting one’s status through self-enhancing humor. Because psychopathic traits have also been linked to adaptive features such as leadership (Lilienfeld et al., 2012) and happiness (Durand, 2018), and narcissism to extraversion (Vernon et al., 2009) and optimism (Hickman et al., 1996), these traits might also predispose to the use of affiliative humor for the sake of self-status enhancement. Conversely, an effective way to lower other people’s status is aggression, that is, behavior intended to harm someone who does not want to be hurt (Bushman and Anderson, 2001). Both reactive (i.e., impulsive) and proactive (i.e., premediated) aggression have been empirically linked to psychopathy (Reidy et al., 2007; Falkenbach et al., 2008) and narcissism (Bushman et al., 2009), as assessed by self-report and behavioral aggression measures. Thus, the use of an aggressive humor style might be predisposed in narcissistic and psychopathic traits.

### Study Aims and Hypotheses

Although our knowledge about the humor correlates of psychopathy and narcissism are vastly improving, three questions remain unaddressed. First, none of the previous studies addressed the triarchic psychopathy components. To address this lacuna, the current study will be the first in the humor area assessing psychopathy with the Psychopathic Personality Inventory-Revised (PPI-R) (Lilienfeld and Widows, 2005). Second, with one exception (Martin et al., 2012), previous studies only examined the relationships between humor and total psychopathy and narcissism scores, preventing sufficient insight into how humor relates to both concepts’ differential subcomponents. Third, previous studies were solely based on raw correlational analyses uncontrolled for the other (sub)factors. To fill these gaps, the current study will use a sufficiently powered sample that allows combining the global psychopathy and narcissism scores along with their subfactors—to provide a fine-grained understanding of the unique contribution of the triarchic psychopathic and four-level narcissistic subcomponents to humor. The first aim of this study is to assess how psychopathy and its subfactors relate to humor, and the second aim to assess how narcissism and its subfactors relate to humor. We generally expect both sets of traits to relate to increased use of self-enhancing and affiliative humor, as well as aggressive humor styles.

## Materials and Methods

### Participants

The sample consisted of *N* = 177 male participants recruited at Maastricht University. The average age was 28.08 years, *SD* = 12.56, 18–68. Only males were included to avoid confounding gender or floor effects because males tend to demonstrate higher levels of psychopathic traits (e.g., Lilienfeld and Andrews, 1996) and narcissism (e.g., Foster et al., 2003). Seventy-five percent of participants were Dutch, 18.5% German, and 6.2% were of other nationalities. Sixty-six percent were students, 22.8% employed, 4.7% work-seeking, and 6.5% were otherwise engaged. About half (53.1%) of participants highest completed education was high school or low-level vocational, 14.7% secondary, and 29.97% high-level, while 2.3% did not disclose their status.

### Measures

#### Humor

Humor styles were assessed using the 32-item Humor Style Questionnaire (HSQ) (Martin et al., 2003; Dutch version: Dikkers et al., 2011), which measures affiliative, self-enhancing, self-defeating, and aggressive humor styles. Each item has to be rated on a seven-point Likert scale ranging from 1 (*totally disagree*) to 7 (*totally agree*). The HSQ has displayed adequate internal consistencies for each of the subscales ranging from α = 0.76 to 0.83 for both the English and the Dutch version (Martin et al., 2003; de Graaf, 2014). Further, it had good test-retest correlations and low intercorrelations between the scales indicating distinct dimensions (Martin et al., 2003). The HSQ’s construct validity was evidenced by positive correlations between the affiliative/self-enhancing scales and life satisfaction and negative correlations with depressive symptoms, while it’s self-defeating and aggressive subscales demonstrated these relationships inversely. Additionally, the HSQ predicted well-being over and beyond the effects of neuroticism (Dyck and Holtzman, 2013). The HSQ’s construct validity has been previously challenged though when, for example, its incremental value to psychological well-being reduced once personality traits (big five inventory) and humor context were accounted for Ruch (2013). Using the current sample, internal consistency levels were α = 0.61 for affiliative, 0.76 for self-enhancing, and 0.74 for both the self-defeating and aggressive humor HSQ subscales.

#### Psychopathy

Psychopathic traits were assessed using the 154-item PPI-R (Lilienfeld and Andrews, 1996; Lilienfeld and Widows, 2005; Dutch version: Uzieblo et al., 2006), which is answered using a four-point Likert scale (1 = *false* to 4 = *true*). Total PPI scores reflect a global index of psychopathy and includes eight subscales. Factor analytic studies revealed a three-factor conceptualization: fearless-dominance (FD), self-centered impulsivity (SCI), and cold-heartedness (Cold) (English: Benning et al., 2003; Dutch: Uzieblo et al., 2010). Previously, FD and SCI have been established as the PPI-R’s main components comprising seven out of eight subscales. Cold-heartedness did not load on either of these two scales, making up the third factor. This three-factor model is in line with the triarchic model of psychopathy (Patrick et al., 2009). The PPI-R has previously demonstrated good external validity (Benning et al., 2003; Uzieblo et al., 2010) high internal consistency (Uzieblo et al., 2010; Hall et al., 2014), and test-retest reliability (Sandler, 2007). The PPI-R’s factor structure has shown a tighter relationship to the Hare Psychopathy Checklist-Revised (PCL-R; Hare, 1991), the golden standard in clinical psychopathy assessment (Poythress et al., 2010), and outperformed the psychometric properties of the Levenson Self-Report Psychopathy Scale (LSRP) in (non)clinical samples (Falkenbach et al., 2007). Using the current sample, internal consistency levels were α = 0.90 for total PPI-R, and 0.91 for the FD, 0.87 for the SCI, and 0.75 for the Cold PPI-R subscales.

#### Narcissism

Grandiose narcissism was assessed with the 37-item Narcissistic Personality Inventory (NPI) (Raskin and Hall, 1979; Morf and Rhodewalt, 1993; Dutch version: Barelds and Dijkstra, 2010). The items are rated on a seven-point Likert scale (1 = *strongly disagree* to 7 = *strongly agree)*. According to Emmons (1984); Emmons (1987) a four-factor solution is most optimal, consisting of superiority and arrogance (SUA), self-absorption and self-administration (SEA), exploitativeness and entitlement (EE), and leadership and authority (LA). The NPI has shown good construct validity (Raskin and Terry, 1988; Barelds and Dijkstra, 2010), good internal (sub)scale reliabilities (Emmons, 1987; Brown and Zeigler-Hill, 2004), and high test-retest correlations (del Rosario and White, 2005). Using the current sample, internal consistency levels were α = 0.89 for total NPI, and 0.66 for the SUA, 0.80 for the SEA, 0.71 for the EE, and 0.84 for the LA NPI subscales.

### Procedure

Participants were recruited using flyer advertisement and convenience sampling. Following written consent, questionnaires were administered on a computer and presented in a random order. Native Dutch participants conducted the study in Dutch, and other participants conducted the study in English. After completing the research, the participants were debriefed, thanked, and either received course credits or a financial compensation. This study was approved by the ethics committee of Maastricht University, reference numbers 114_06_05_2012, 2014_04_28_08_42_15, and 145_07_10_2014.

### Statistical Analyses

All analyses were conducted using SPSS version 24 (IBM Corp, 2016). Correlations between the study variables were assessed using Pearson correlations. Regression analyses were conducted to assess the relationships between humor and the psychopathy and narcissism subscales. Here, the humor styles served as the dependent variable, while the psychopathy and narcissism subscales were predictors. To address the first research aim, each humor style was assessed separately by means of multiple regressions enter-method with the PPI-R subfactors as simultaneous predictors. To address the second research aim, the same was done with the NPI subfactors as predictors.

## Results

### Descriptive Results

Descriptive statistics and correlation analyses can be seen in Table 1. Significant positive correlations between all styles of humor, except between self-enhancing and self-defeating humor, were observed^1^.

**TABLE 1:** Mean, standard deviations and intercorrelations among study variables (*N* = 177).

**Variables**	***M (SD)***	**1**	**2**	**3**	**4**
1. HSQ affiliative	5.36 (.70)	1			
2. HSQ self-enhancing	4.85 (.90)	0.41**	1		
3. HSQ self-defeating	3.51 (.95)	0.30**	0.12	1	
4. HSQ aggressive	4.04 (1.05)	0.35**	0.19*	0.35**	1
5. PPI-R total	300.36 (30.38)	0.36**	0.28**	0.08	0.39**
6. PPI-R FD	123.75 (18.66)	0.31**	0.32**	−0.10	0.11
7. PPI-R SCI	141.79 (19.67)	0.31**	0.12	0.29**	0.47**
8. PPI-R Cold	34.81 (6.24)	−0.14	0.04	−0.23**	0.09
9. NPI total	154.00 (23.02)	0.35**	0.21**	0.17*	0.34**
10. NPI-SUA	43.36 (7.19)	0.25**	0.24**	0.13	0.20**
11. NPI-SEA	40.41 (7.76)	0.32**	0.16*	0.14	0.25**
12. NPI-EE	28.57 (6.87)	0.23**	0.06	0.29**	0.42**
13. NPI-LA	41.66 (8.05)	0.27**	0.18*	−0.00	0.19*

PPI-R FD, Fearless Dominance; PPI-R SCI, Self-centered Impulsivity; PPI-R Cold, Cold-heartedness; NPI-SUA, Superiority and Arrogance; NPI-SEA, Self-absorption and Self-administration; NPI-EE, Exploitativeness and Entitlement; NPI-LA, Leadership Authority. *p < 0.05; **p < 0.001.

### Aim 1: Relationship Between Humor and Psychopathic Traits

Affiliative humor was positively correlated with all psychopathic variables except for PPI-R-Cold. Self-enhancing humor was significantly associated with PPI-R-total and PPI-R-FD. Self-defeating humor was positively correlated with PPI-R-SCI and negatively with PPI-R-Cold. Aggressive humor exhibited positive significant relations with PPI-R-total and PPI-R-SCI (Table 1).

The regression analysis using all PPI subscales, revealed significant models for affiliative (*F* = 15.61, *p* < 0.001, *R*^2^ = 0.21), self-enhancing (*F* = 6.89, *p* < 0.001, *R*^2^ = 0.11), self-defeating (*F* = 11.18, *p* < 0.001, *R*^2^ = 0.16), and aggressive humor (*F* = 16.47, *p* < 0.001, *R*^2^ = 0.22). Affiliative humor was significantly positively predicted by PPI-R-FD and PPI-R-SCI, and negatively by PPI-R-Cold. PPI-R-FD was the only positive significant predictor of self-enhancing humor. Self-defeating humor was significantly positively associated with PPI-R-SCI and negatively with PPI-R-Cold. PPI-R-SCI was a significantly positively related to aggressive humor (Table 2).

**TABLE 2:** Results of the four sets of multiple regression analyses with humor styles as dependent variables, and separately psychopathy total, narcissism total, psychopathy subscales, and narcissism subscales as independent variables (*N* = 177).

	**Affiliative Humor**			**Self-Enhancing Humor**			**Self-Defeating Humor**			**Aggressive Humor**		
	***ß***	***t***	***p***	***ß***	***t***	***p***	***ß***	***t***	***p***	***ß***	***t***	***p***
*Psychopathy*												
PPI-R total	0.36**	5.13	<0.001	0.28**	3.85	<0.001	0.08	1.12	0.27	0.39**	5.57	<0.001
PPI-R-FD	0.30**	4.36	<0.001	0.31**	4.22	<0.001	–0.11	–1.46	0.15	0.05	0.71	0.48
PPI-R-SCI	0.30**	4.34	<0.001	0.08	1.09	0.28	0.33**	4.70	<0.001	0.46**	6.75	<0.001
PPI-R-Cold	−0.21**	–3.08	0.002	–0.01	–0.12	0.91	−0.25**	–3.47	0.001	0.03	0.49	0.62
*Narcissism*												
NPI total	0.35**	4.89	<0.001	0.21**	2.81	0.01	0.17*	2.30	0.02	0.34**	4.73	<0.001
NPI-SUA	0.06	0.64	0.52	0.23*	2.37	0.02	0.07	0.77	0.44	–0.05	–0.51	0.61
NPI-SEA	0.21*	2.40	0.02	0.10	1.06	0.29	0.05	0.56	0.58	0.06	0.72	0.48
NPI-EE	0.06	0.73	0.47	–0.11	–1.24	0.22	0.28**	3.23	0.001	0.40**	4.78	<0.001
NPI-LA	0.12	1.27	0.21	0.04	0.37	0.71	–0.16	–1.74	0.08	0.06	0.61	0.54

PPI- R-FD, Fearless Dominance; PPI-R-SCI, Self-centered Impulsivity; PPI-R-Cold, Cold-heartedness; NPI-SUA, Superiority and Arrogance; NPI-S-EA, Self-absorption and Self-administration; NPI-EE, Exploitativeness, and Entitlement; NPI-LA, Leadership and Authority. *p <.05; **p <.01.

### Aim 2: Relationship Between Humor and Narcissistic Traits

Affiliative and aggressive humor positively correlated with all narcissism variables. Self-enhancing humor was significantly associated with total narcissism and the subscales of SUA, SEA, and LA. Moreover, self-defeating humor was positively correlated with NPI-total and NPI-EE (Table 1).

The multiple regression analysis with all NPI subscales demonstrated a significant model for affiliative humor (*F* = 6.28, *p* < 0.001, *R*^2^ = 0.13), in which NPI-SEA served as the positive predictor. Self-enhancing humor revealed a significant model (*F* = 3.30, *p* < 0.05, *R*^2^ = 0.07) and was significantly positively predicted by NPI-SUA. The models for self-defeating (*F* = 4.63, *p* < 0.001, *R*^2^ = 0.10) and aggressive humor (*F* = 9.61, *p* < 0.001, *R*^2^ = 0.18) were significant, and NPI-EE positively and significantly predicted both these humor styles (Table 2).

## Discussion

This study investigated humor styles in two dark forms of psychopathological traits that negatively impact interpersonal relationships. Specifically, the focus was on subclinical levels of (subcomponents of) psychopathy and grandiose narcissism. Overall, psychopathic and narcissistic traits related to various benign and injurious humor styles, directed toward others and the self.

Regarding the first study aim, as expected, total psychopathy scores showed to relate to affiliate, self-enhancing, and aggressive humor in a positive, and largely medium-strong, way. The link between psychopathy and aggressive humor styles is in line with the fact that excessive aggression levels are reflected in several psychopathy criteria (Hare, 1991), and with experimental research evidencing a positive relationship between psychopathy and both self-reported (Falkenbach et al., 2008; Cima and Raine, 2009) and behavioral aggression (Reidy et al., 2007). This was further evidenced by our finding that the psychopathy subfactor of self-centered impulsivity drove this relationship, basically reflecting disinhibited antisocial behavior. This finding implies that the psychopathic personality components alone may not be sufficiently related to the use of aggressive humor, but in addition, this use may be provoked by a lack of self-control. This study was the first to find such clear evidence for a positive relationship between psychopathy and affiliative humor. Analyses of the psychopathy subscales showed that this relationship was driven by the fearless-dominance and self-centered impulsivity factors, suggesting that the boldness and the disinhibition aspects of psychopathy relate to an outgoing and carefree attitude including a tendency to connect with others through humor. Regarding self-enhancing humor, our data reveal that psychopathic tendencies in general, but particularly boldness, equip people with the ability to cope with stressors through humor. The positive and unique relationship between self-defeating humor and the self-centered impulsivity psychopathy factor bears a resemblance to Martin et al. (2012) who linked this humor style to the erratic lifestyle psychopathy component. Even though Martin et al. (2012) utilized a different psychopathy instrument [i.e., the Self-Report Psychopathy (SRP) Scale], their measurement follows the same factor structure as the PPI-R used in our sample (Benning et al., 2005; Derefinko and Lynam, 2006). Hence, both results suggest that especially those high in disinhibition employ self-disparaging humor to create bonds with others. Finally, the humor correlates of the cold-heartedness component of psychopathy—indicating affective detachment and meanness—distinctively deviates from the other psychopathy factors. Specifically, cold-heartedness was inversely related to both affiliative and self-defeating humor styles. This is in line with evidence pinpointing cold-heartedness as a unique determinant associated with a lack of emotionality and negative relations to the big five personality traits (especially agreeableness) (Berg et al., 2015) and a reduced requirement for personal space (Vieira and Marsh, 2014). Moreover, cold-heartedness has been identified as a specific risk factor for extremely devastating social behavior, such as heightened proactive criminal thinking (Walters, 2016) and increased bug-killing combined with higher positive affect and decreased guilt (i.e., sadistic pleasure) (Lobbestael et al., 2020). Overall, our results do not fit the conceptualization of psychopathy as a unitary, one-dimensional construct (Marcus et al., 2013). Instead, they support the triarchic conceptualization of psychopathy (Patrick et al., 2009) in which boldness specifically drives the use of self-enhancing humor, and disinhibition fuels the use of aggressive and potentially self-defeating humor styles. In contrast, meanness turns out to be a particular humorless concept. The latter finding highlights that cold-heartedness might be particularly devastating for interpersonal relationships, as it prevents the use of humor to strengthen interpersonal bonds. While our findings on psychopathy and the use of aggressive and self-defeating humor styles largely mimic previous studies, the current study was the first to reveal relationships with affiliative and self-enhancing humor styles. The latter could be attributed to measuring psychopathic traits using the PPI-R. Compared to measures like the LSRP or SRP, the PPI-R has the advantage of assessing the triarchic components of psychopathy and being of superior psychometric quality (Falkenbach et al., 2007).

Regarding the second study aim, global grandiose narcissism scores displayed a positive relationship with all humor styles. The expected relationship with affiliative and aggressive humor was of medium effect size, while the self-directed humor styles represented small effects. In-depth analyses of the different subscales of narcissism revealed that two subscales drove the use of benign humor styles. Specifically, self-absorption/self-admiration traits predisposed to using humor to bond with others (i.e., affiliative), and superiority and arrogance traits increased the inclination to cope with difficult situations through humor (i.e., self-enhancing). In contrast, the exploitative and entitlement scale can be considered the most maladaptive aspect of narcissism, related to increased use of both injurious humor styles. There has been abundant empirical evidence linking narcissism to aggression, for example, through ego-threat (Bushman et al., 2009). Similar to previous studies (Zeigler-Hill and Besser, 2011; Martin et al., 2012), the current study suggests that this link could plausibly be further deepened by the use of aggressive humor, to put down or manipulate others. Although unpredicted in this study, narcissism has been shown to relate to self-defeating humor before (Besser and Zeigler-Hill, 2011; Zeigler-Hill and Besser, 2011). This could be reflecting the self-critical or low self-esteem aspect assumed to underlie narcissists’ grandiose overt display (Jordan et al., 2003; Young et al., 2003). Our study infers that self-defeating humor might be instrumentally installed as an exploitative means to create bonds with others among those scoring high in narcissism.

Strengths of the current study include the investigation of how humor relates to the triarchic components of psychopathy and stricter analyses of the differential subfactors of psychopathy and narcissism. In addition, this is the first study on humor tendencies using a European sample, which might be relevant given the cultural differences in the meaning and expression of humor (Kalliny et al., 2006). Limitations include the use of a male sample, preventing generalization to females. This might have impacted our findings on aggressive humor styles, which might be used and appreciated more frequently by males (Kotthoff, 2006). Future studies are needed to assess how gender might differentially impact the link between humor and personality traits like psychopathy and narcissism. Another limitation is the reliance on self-reports of humor in this study. Especially, the use of aggressive humor might be underreported because of social desirability. Furthermore, the internal consistency level of the affiliate humor style was lower than that in other studies (e.g., Martin et al., 2003; Ruch and Heintz, 2017), and might have been due to the combination of a two-language sample. Lastly, given that the construct validity of the HSQ is sometimes challenged due to its possible overlap with personality traits (Ruch, 2013), it would be interesting for future studies to compare the incremental value of adding Big Five personality traits while investigating the relationship between psychopathic/narcissistic traits and humor. Moreover, future research should additionally examine the relation between psychopathic and narcissistic traits and humor using, for example, the Humor-Behavior Q-Sort Deck (Craik et al., 1996), to avoid the HSQ’s uncertainties regarding its items’ context (Ruch, 2013; Ruch and Heintz, 2017).

The current study highlights the importance of humor in two of the most devastating types of psychopathologic traits. The fact that both sets of traits have shown to be linked to benign humor styles comes with potential societal implications. First, the link with affiliative humor shows that both psychopathic and narcissistic traits are related to an intact, and even superior use of humor to create interpersonal bonds. Therefore, humor could have the potential to serve as a tool to strengthen and repair interpersonal relationships in people with elevated psychopathic and narcissistic traits—this is especially conceivable in case they would be able to succeed in simultaneously lowering their use of aggressive humor. Second, encouraging self-enhancing humor could potentially be beneficial because it has shown to be effective in coping with stress (Abel, 2002; Martin, 2006). In sharp contrast, the use of injurious humor styles should be discouraged and be taken seriously as a possible indication of undermining one’s self-image and one’s relationships with significant others. A first step could be to raise the awareness of people with higher levels of psychopathy or narcissism regarding the probable devastating and perpetuating impact of using self-defeating or aggressive humor styles and assist them in bending injurious humor styles to more adaptive interaction styles. In case future studies evidence a causal link between humor and psychopathy/narcissism in a clinical sample, therapists might be sensitized to the increased use of humor in therapeutic relationships with psychopathic and narcissistic clients, as a potential tool to create and strengthen the therapeutic relationship. Humor has been shown to positively affect both treatment outcomes and treatment satisfaction for patients and therapists (Kidd et al., 2009). Therefore, it is not surprising that several therapeutic strategies pinpoint the use of humor as an effective therapeutic tool, for example, through strengthening the formation of the Happy Child mode in Schema Therapy (Young et al., 2003).

Taken together, the current, fine-grained analyses of psychopathic and narcissistic (sub)traits evidenced that humor is a frequent and important correlate of both these personality constellations. The two injurious humor styles (i.e., aggressive and self-defeating) were mostly associated with impulsivity and entitlement. Dominance levels seemed to actuate the use of humor as a stress-coping method. Using humor for affiliative means was linked to a broader range of psychopathic and narcissistic traits. The cold-heartedness component of psychopathy clearly lacks humor which, again, stresses its particularly disturbing nature.

## Data Availability Statement

The raw data supporting the conclusions of this article will be made available by the authors, without undue reservation.

## Ethics Statement

The studies involving human participants were reviewed and approved by The Ethics Review Committee Psychology and Neuroscience (ERCPN). Written informed consent to participate in this study was provided by the participants.

## Author Contributions

JL was responsible for study conceptualization and data collection. JL and VF were responsible for data preparation, data analysis, and report writing. Both authors contributed to the article and approved the submitted version.

## Conflict of Interest

The authors declare that the research was conducted in the absence of any commercial or financial relationships that could be construed as a potential conflict of interest.

## Footnotes

1 The intercorrelations between the PPI-R and the NPI subscales are reported in the Supplementary Table.
