# Supplementary material for: Humor in Dark Personalities: An Empirical Study on the Link Between Four Humor Styles and the Distinct Subfactors of Psychopathy and Narcissism
Source: Front Psychol. 2021 Apr 12;12:548450. doi: 10.3389/fpsyg.2021.548450 (PMC8072024; doi:10.3389/fpsyg.2021.548450)
Supplement: Supplementary file 1 [file Table_1.pdf]

Supplementary table: Intercorrelations between the PPI-r and the NPI subscales

	NPI total	NPI SUA	NPI SEA	NPI EE	NPI LA
PPI-R total	.55**	.44**	.41**	.34**	.50**
PPI-R FD	.47**	.42**	.38**	.06	.55**
PPI-R SCI	.39**	.25**	.28**	.46**	.24**
PPI-R Cold	.05	.11	-.01	.03	.03

Note: PPI-R FD = Fearless Dominance; PPI-R SCI = Self-centred Impulsivity; PPI-R Cold = Cold-heartedness; NPI-SUA = Superiority & Arrogance;

NPI-SEA = Self-absorption & Self-administration; NPI-EE = Exploitiveness & Entitlement; NPI-LA = Leadership Authority. * $p < .05$; ** $p < .001$.
